# Electroacupuncture ameliorating post-stroke cognitive impairments via inhibition of peri-infarct astroglial and microglial/macrophage P2 purinoceptors-mediated neuroinflammation and hyperplasia

**DOI:** 10.1186/s12906-017-1974-y

**Published:** 2017-10-10

**Authors:** Jia Huang, Xiaofang You, Weilin Liu, Changming Song, Xiaomin Lin, Xiufeng Zhang, Jing Tao, Lidian Chen

**Affiliations:** 1 College of Rehabilitation Medicine, Fujian University of Traditional Chinese Medicine, Fuzhou, Fujian 350122 China; 2 Fujian Key Laboratory of Rehabilitation Technology, Fuzhou, Fujian 350122 China

**Keywords:** Electroacupuncture, Ischemic stroke, Neuroinflammation, Glia, P2 purinoceptors

## Abstract

**Background:**

During ischemic stroke (IS), adenosine 5′-triphosphate (ATP) is released from damaged nerve cells of the infract core region to the extracellular space, invoking peri-infarct glial cellular P2 purinoceptors singling, and causing pro-inflammatory cytokine secretion, which is likely to initiate or aggravate motor and cognitive impairment. It has been proved that electroacupuncture (EA) is an effective and safe strategy used in anti-inflammation. However, EA for the role of purine receptors in the central nervous system has not yet been reported.

**Methods:**

Ischemia-reperfusion injured rat model was induced by middle cerebral artery occlusion and reperfusion (MCAO/R). EA treatment at the DU 20 and DU 24 acupoints treatment were conducted to rats from the 12 h after MCAO/R injury for consecutive 7 days. The neurological outcomes, infarction volumes and the level of astroglial and microglial/macrophage hyperplasia, inflammatory cytokine and P2X7R and P2Y1R expression in the peri-infarct hippocampal CA1and sensorimotor cortex were investigated after IS to evaluate the MCAO/R model and therapeutic mechanism of EA treatment.

**Results:**

EA effectively reduced the level of pro-inflammatory cytokine interleukin-1β (IL-1β) as evidenced by reduction in astroglial and microglial/macrophage hyperplasia and the levels of P2X7R and ED1, P2X7R and GFAP, P2Y1R and ED1, P2Y1R and GFAP co-expression in peri-infarct hippocampal CA1 and sensorimotor cortex compared with that of MCAO/R model and Non-EA treatment, accompanied by the improved neurological deficit and the motor and memory impairment outcomes. Therefore, our data support the hypothesis that EA could exert its anti-inflammatory effect via inhibiting the astroglial and microglial/macrophage P2 purinoceptors (P2X7R and P2Y1R)-mediated neuroinflammation after MCAO/R injury.

**Conclusion:**

Astroglial and microglial/macrophage P2 purinoceptors-mediated neuroinflammation and hyperplasia in peri-infarct hippocampal CA1 and sensorimotor cortex were attenuated by EA treatment after ischemic stroke accompanied by the improved motor and memory behavior performance.

**Electronic supplementary material:**

The online version of this article (10.1186/s12906-017-1974-y) contains supplementary material, which is available to authorized users.

## Background

Electroacupuncture (EA) is an effective and safe strategy used for anti-inflammation and anti-nociception of many diseases [1–3]. Solid evidence of purinergic signaling mediating the peripheral functional mechanisms underlying analgesic EA has been demonstrated [4, 5]. EA induces the release of extracellular ATP in turn activates purinergic receptors on the sensory nerves of the skin, then transmits those messages to the spinal cord nerve root and higher brain centers that control autonomic functions and modulate neural activities [4–7]. However, EA acts on purine receptors in the central nervous system(CNS) has not yet been reported.

It is well-documented that extracellular ATP triggers surrounding glial purinergic receptors signaling pathway and pro-inflammatory cytokines release to aggravate neural injury in cerebral ischemia [8, 9]. Therefore, we speculate that purinergic receptors might play dualistic roles in response to EA effects treating inflammatory injury induced by ischemia.

Our previous studies have demonstrated that EA protects cerebral neural cells against inflammatory injury after cerebral ischemia, which appears at 24 h to 14 days after treatment. It can also suppress the nucleus translocation of nuclear factor kappa b (NF-κB) p65 to attenuate pro-inflammatory cytokine transcription and secretion in cerebral glial cells, which elicits protective effects against ischemic injury [10–12]. The upstream extracellular and membrane signal molecules which transmit EA effects to NF-κB-p65 remains unknown.

It is reported that NF-κB is activated by P2X purinoceptor 7(P2X7R) and P2Y purinoceptor 1(P2Y1R) in inflammatory reactions, whereas lack of a functional P2X7R leads to a reduction of NF-κB translocation [13–16]. However, the P2X7R/P2Y1R related EA treatment with the development of cerebral ischemic injury is complex and unclear.

P2 purinoceptors are a family of plasma membrane molecules, in which P2X7R is an ATP-gated ion channel, while P2Y1R is a G protein-coupled receptor [17]. The P2 purinoceptors have been play an important role in cell-to-cell communication, pro-inflammatory cytokine release, microglial/macrophage activation and proliferation [18, 19]. Microglia is one of the immunoeffector cells of the CNS, it is activated rapidly after ischemia and produces pro-inflammatory mediators and neurotoxic compounds which exacerbates neuroinflammation and secondary brain injury [20]. Studies have suggested that ischemic injury could exhibit enhanced P2X7R expression in the neuroinflammatory area where activated microglia is a coexisting feature [21–23]. Inhibition of microglial P2X7R, co-localized with the microglial marker Iba-1, relieves enhanced cerebral edema and neurological injury after brain injury in mice [24, 25]. Extracellular ATP stimulates astrocytic/macrophage P2Y1R, resulting in high expression, leading to transcription of inflammatory genes [15]. A number of evidences have suggested that inhibition of activated microglial and pro-inflammatory release is involved in EA treatment for brain injury [10, 26]. A well-documented finding displays that EA could suppress P2 purinoceptors activity to inhibit in spinal microglia activation for relieving nerve injury-induced pain hypersensitivity [27].

Based on these findings, it can be speculated that inhibition of glial/macrophage purinoceptors is considered as an important therapeutic strategy for effectively preventing pro-inflammatory cytokines release and protecting cerebral neurological injury after ischemic stroke. Therefore, the present study proposed a hypothesis that EA could alleviate ischemic injury-induced neuroinflammation and brain injury via inhibition of peri-infarct astroglial and microglial/macrophage P2 purinoceptors function.

## Methods

### Animals and ethics

All the experiments in this study were in strict accordance with the Provision and General Recommendation of Chinese Experimental Animals Administration Legislation and were approved by the Animal Ethics Committee of Fujian University of Traditional Chinese Medicine (Fuzhou, China). Adult male Sprague-Dawley rats (weight, 240-270 g) were obtained from Shanghai SLAC Laboratory Animal Co. Ltd. (Shanghai, China, SCXK2013–0005), and housed under pathogen-free conditions with a 12 h/12 h light/dark cycle at the Experimental Animal Center of Fujian University of Traditional Chinese Medicine, in groups of five per cage.

### Experimental procedures

Ischemia-reperfusion injured rat model was established by middle cerebral artery occlusion and reperfusion (MCAO/R) as previously described [28, 29]. Focal cerebral ischemia was monitored using transcranial temporal laser Doppler (BIOPAC Systems, Goleta, CA, USA) and an 80% decrease in blood flow after the occlusion was recorded. After 90 min of occlusion, reperfusion was achieved by pulling out the filament to restore blood flow. Rats were randomly assigned to four groups according to the random number table (*n* = 12 rats each group) as follows: (1) Sham group, (2) MCAO/R group, (3) MCAO/R + EA group, and (4) MCAO/R+ Non-EA group. The Sham group underwent the same procedure, but arterial occlusion was not performed. After 24 h of MCAO/R surgery, the Sham group and the MCAO/R group received no treatment, while MCAO/R + EA group received EA treatment using stainless steel acupuncture needles (outer diameter 0.3 mm, length 3 mm) at the acupoints of Baihui (DU20, located in the median of the parietal bone and the line linking the two ears.) and Shenting (DU24, located in the median of frontalis) with an inserted depth of 2–3 mm, stimulated with the intensity of 0.2 mA, peak voltage of 6 V, and dilatational waves of 2/20 Hz using the electronic acupuncture treatment instrument (Model G6805; SMIF, Shanghai, China), 30 min per day for 7 consecutive days. The MCAO/R+ Non-EA group were given EA treatment at the non-acupoints (located in the costal region and 10 mm distal to the iliac crest), and the EA needles and stimulation parameters of the bilateral non-acupoints were consistent with the EA treatment [30] Additional file 1.

### Neurobehavioral assessment

Neurobehavioral functional tests were assessed before EA treatment and at days 1, 3 and 7 after treatment, by two researchers who were blinded to the experimental groups. The neurological deficits were determined according to Longa’s scoring system [28], as follows: score 0, no neurological deficits; score 1, (failure to fully extend the right forepaw) mild deficits; score 2 (circling to the right) and score 3(falling to the right) moderate deficits; score 4, (cannot walk spontaneously and has depressed levels of consciousness) severe deficits. Rats scoring 0 or 4, exhibiting either no or severe deficits, respectively, were excluded from the current study. The higher the neurological deficit score, the more severe the impairment is of motor motion injury.

The modified neurological severity scores (mNSS) which included a composite of motor, sensory, reflex, and balance tests, with scores ranging from 0 to 18 (normal score: 0; maximal deficit score: 18) was conducted [31]. Rats showed 7–13 points in the mNSS immediately after reperfusion were included in this study.

### Morris water maze test

At 3 days after EA, all the rats were subjected to the Morris water maze test to evaluate spatial learning and memory. The water maze apparatus (Chinese Academy of Sciences, Beijing, China) consisted of a tank (diameter, 120 cm; height, 50 cm) filled with water (depth, 30 cm; temperature, 25 ± 2 °C). The tank was divided into 4 equal quadrants. A circular escape platform, measuring 6 cm in diameter and 28 cm in height, was submerged 2 cm below the water surface, in the middle of the third quadrant of the pool and the reference objects around the pool were placed. A video camera attached to a computer was placed above the center of the tank for recording and analysis of the rats. These points served as the starting positions at which each rat was lowered gently into the water, its head facing the wall of the water maze. Morris water maze tasks mainly include orientation navigation and space exploration trials. The experiment included two phases:

Phase I. *Acquisition*: Acquisition training consisted of 4-day conditioning with 4 trails per day from day 3 to 6 after ischemia. For each trail, the rat was placed in the water facing the wall of the pool at one of the four starting points (north, south, east or west) and allowed to swim for a maximum of 90 s. If the rat found the platform, it can remain on it for 15 s. If it did not find the platform, it was guided to it and allowed to remain there for 15 s.

Phase II. Probe trails, on day 7, the platform was removed and rats were given one 90 s retention probe test. During retention, the number of times each animal crossed the position in which the platform had previously been located and the time spent in the quadrant were measured.

After all trials, the rats were dried thoroughly with a hair drier and returned to their cages. Morris water maze test was repeated for three times.

### T2-weighted magnetic resonance imaging

Animal MRI scans were performed using a 7.0 T MRI scanner (BioSpec 70/20 USR, Bruker Biospin Gmbls, Germany). After rats were anesthetized with isoflurane (2.5%), their body temperature was controlled with a water circulating heating bed (SA Instruments, Stony Brook, NY) and respiratory rate was monitored during scanning with MRI-compatible small animal gating system (Model 1025, SA Instruments, Stony Brook, NY). T2-weighted images (T2WI) were collected at Days 3 and 7 after EA. T2WI scans were performed using a gradient-echo sequence with following parameters: TR/TE = 4200/35 ms, FOV = 32 × 32 mm, Averages = 2, Matrix = 256 × 256, Slices = 21, Slice Thickness = 0.8 mm. The infarct volume was calculated over all slices (volume of lesion/total brain volume) using Image J and expressed as a percentage of total brain volume.

### Immunohistochemistry and immunofluorescence staining

Immunohistochemistry was performed by methods described previously [32]. The brain slices were incubated with the primary antibodies mouse anti-GFAP (dilution of 1:800, ab7260, Abcam, UK); anti-ED1 (dilution of 1:300, #12389, Cell Signaling Technology, USA) in a humidified chamber. In the subsequent reaction, slices were washed and incubated with biotinylated secondary antibodies followed by Avidin-Biotin-Peroxidase treatment, developed with DAB (DAB Kit-0017, Maixin, China), and counterstained with haematoxylin to visualize cell nuclei. Images were detected by microscope (DM4000LED, Leica, Germany) at 400× and analyzed by investigators blinded to groups using the Image-pro plus analysis system.

For double immunofluorescence analysis, after blocking in 10% (*v*/v) bovine and donkey serum albumin in PBS in the 37 °C incubator for 2 h,the slices were simultaneously incubated overnight at 4 °C with an antibody mixture of antibodies mouse anti-GFAP (dilution of 1:300, ab6326, Abcam, UK), anti-ED1 (dilution of 1:200, ab6326, Abcam, UK), rabbit anti-P2Y1R (dilution of 1:30, Santa Cruz Biotechnology, USA) and rabbit anti-P2X7R (dilution of 1:50, Cell Signaling Technology, USA) in a humidified chamber. After that, slices were washed a minimum of three times (5 min per wash), and incubated with secondary antibodies conjugated to fluorophores Alexa Fluor 546 (donkey anti-rabbit, dilution of 1:100, A10040, Santa Cruz, USA) and Alexa Fluor 488 (donkey anti-mouse, dilution of 1:100, A21202, Life Technologies, USA), for 2 h at 37 °C in the dark moist chamber. After the sections were washed with PBS, they were counterstained with DAPI (dilution of 1:1000; Santa Cruz, USA) and re-suspended in mounting medium (Vector Laboratories, USA). Images were captured by a confocal fluorescence microscope (LSM710, Carl Zeiss, Germany) at 400× magnification. Omission of the primary antibody served as the negative control for all experiments and showed no staining.

### Enzyme-linked immunosorbent assay (ELISA)

Tissue homogenates of the ipsilateral hippocampus and sensorimotor cortex were separated by centrifugation for 20 min at 3000 r.p.m. Inflammatory cytokines IL-1β and IL-10 levels were detected by ELISA kits according to the manufacturer’s protocols (Xitang, Shanghai, China). In brief, samples were coated with 100 μl capture antibody at 4 °C. Followed three washes, using 200 μl assay diluents to block at room temperature for 1 h, then 100 μl diluted IL-1β or IL-10 standards and test samples were added and incubated for 1 h at 37 °C. Following repeated washes, each well was added with the substrate and incubated for 20 min at room temperature. The quantitative absorbance was determined at 450 nm using an ELISA reader (BioTek, Model ELX800, USA) according to IL-1β or IL-10 protein standards.

### Statistical analysis

All data were presented as mean ± standard error of the mean (S.E.M) from at least 6 animals per group. The significance of differences between groups was examined using one-way analysis of variance (ANOVA) and Student’s t-tests. The homogeneity of variance was analyzed using the least significant difference method and missing variance using the Games-Howell method. At least three independent experiments were carried out. *P* value of less than 0.05 were considered significant. All final results were analyzed in a blinded manner.

## Results

### EA treatment improved neurological deficit and motor function in MCAO/R rats

**Fig. 1:**
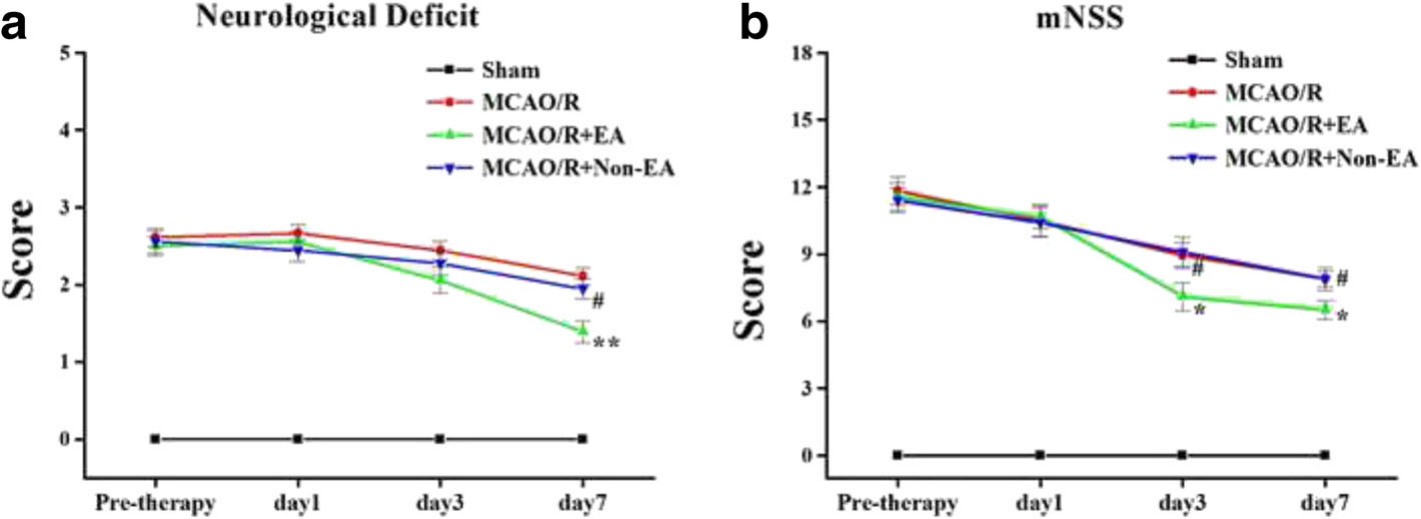
Neurological functional outcome. **a**, **b** The Data of neurological deficit score and mNSS score are shown as mean ± S.E.M from 12 individual rats in each group. *P < 0.05, **P < 0.01, the MCAO/R + EA group versus the MCAO/group; ^#^ *P* < 0.05, the MCAO/R+ Non-EA group versus the MCAO/R + EA group

To evaluate effects of EA treatment-mediated neuroprotection in MCAO/R rats, the neurological deficit score and the mNSS test were performed to assess the neurological function of the rats in each group before treatment and at Days 1, 3, and 7 after EA. Rats in the Sham group showed no motor or behavioral impairments. There was no significant difference in neurological deficit score and mNSS among the MCAO/R group, the MCAO/R + EA group and the MCAO/R+ Non-EA group before EA treatment (*P* > 0.05, Fig. 1a and b). However, these neurological scores were ameliorated in the MCAO/R + EA group at Day 7 after EA treatment, as compared with the MCAO/R group (*P =* 0.001, Fig. 1a) and the MCAO/R+ Non-EA group (*P =* 0.013, Fig. 1a). The mNSS scores were ameliorated in the MCAO/R + EA group at Days 3 and 7 after EA treatment, as compared with the MCAO/R group (Day3: *P =* 0.044, Day7: *P =* 0.027, Fig. 1b) and the MCAO/R+ Non-EA group (Day3: *P =* 0.031, Day7: *P =* 0.032, Fig. 1b), These results demonstrated that EA could produce neuroprotective effects against neurological and motor deficits induced by MCAO/R.

### EA treatment ameliorated cognitive impairment in MCAO/R rats

**Fig. 2:**
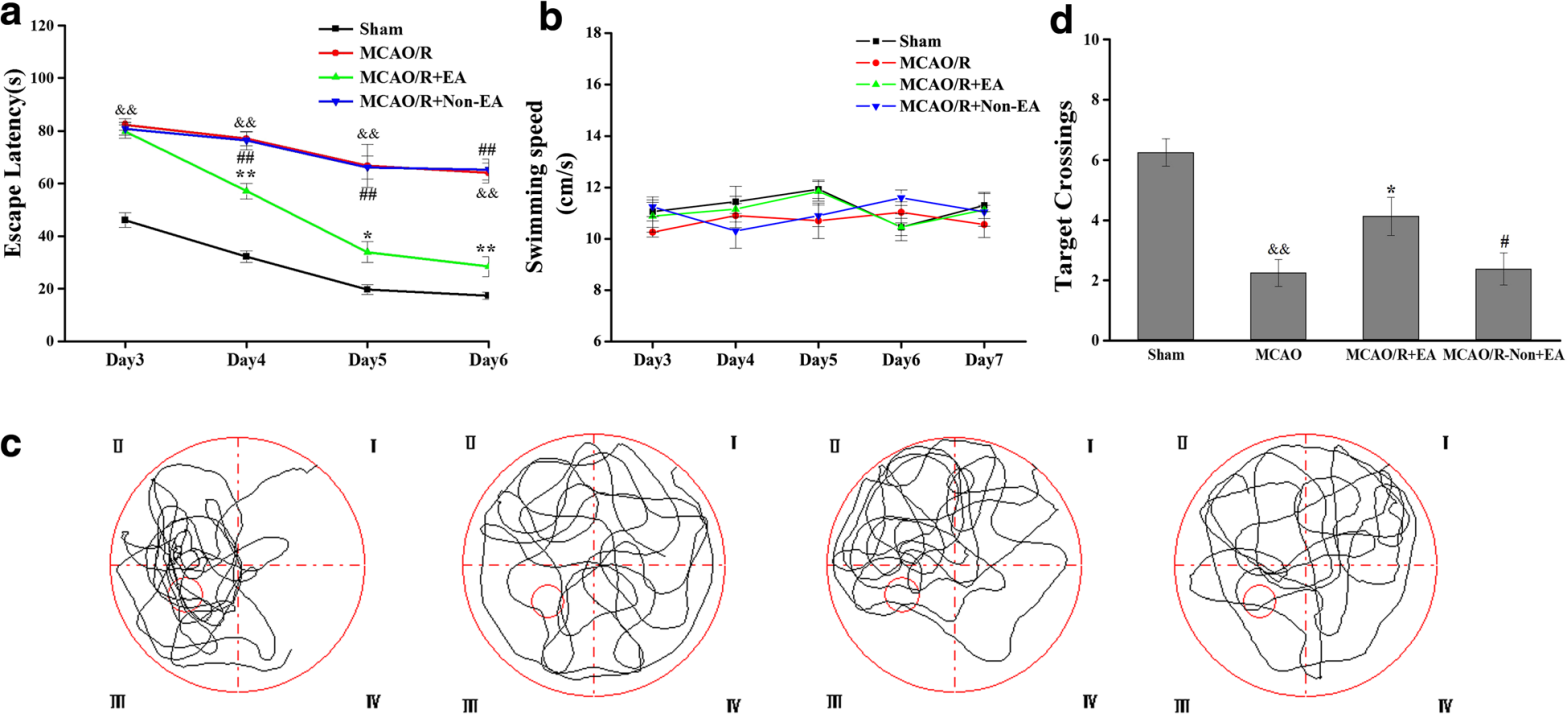
Evaluation of spatial reference learning and memory by Morris Water Maze test. **a**, **b** Mean escape latency time and swimming speed during the orientation navigation test on Days 3–6 after EA treatment. **c**, **d** Times the rats crossed over platform location and representative path diagrams on Day 7 after EA treatment during the spatial memory test in different groups. (*n* = 12 each group; ^&&^ *p* < 0.01, the MCAO/R group versus the Sham group; **P < 0.01, *P < 0.05, the MCAO/R + EA group versus the MCAO/group; ^##^ *P* < 0.01, ^#^ *P* < 0.05, the MCAO/R+ Non- EA group versus the MCAO/R + EA group

All rats were assessed in a Morris water maze test on days 3–7 after EA treatment. Spatial learning was assessed by the time required to find the hidden platform (escape latency). The latency to reach the hidden platform in the maze was significantly increased in the MCAO/R group compared with the Sham group (*P* < 0.01, Fig. 2a), whereas the times that rats crossed the platform’s location were significantly decreased compared with rats in the sham group (*P* < 0.05, Fig. 2d). The swimming traces of Sham-operated and EA-treated rats concentrated in the target quadrant where the platform had been set. However, the swimming traces of the MCAO/R and Non-EA group rats were uniformly distributed around four quadrants (Fig. 2c). These indicates that cerebral MCAO/R injury resulted in cognitive impairment. However, EA significantly decreased the latency compared with the MCAO/R group (Day 4: *P* < 0.001, Day 5: *P* = 0.021, Day 6: *P* < 0.001, Fig. 2a) and the MCAO/R+ Non-EA group(Day 4: *P* < 0.001, Day 5: *P* < 0.001, Day 6: *P* < 0.001, Fig. 2a), and increased the number of target crossings compared with the MCAO/R group (*P =* 0.018, Fig. 2d) and the MCAO/R+ Non-EA group (*P =* 0.026, Fig. 2d). Therefore, EA treatment could ameliorate ischemia-induced spatial learning and memory impairment. To exclude the possibility that the improvement of EA on cognitive impairment in the MCAO/R rats was not due to sensorimotor abnormalities, we analyzed their swimming ability. However, there was no difference in swimming speed among the groups of rats across the four training days and testing day (*p* > 0.05, Fig. 2b), suggesting that the impaired spatial cognitive ability of ischemic rats in the water maze test is not caused by motor deficits. Additionally, we performed a visible cued task to exclude abnormal basic learning or visual acuity problems among the rats. The groups of rats did not exhibit any impairment in cue-task performance (data not shown).

### EA treatment reduced cerebral infarct volume in MCAO/R rats

**Fig. 3:**
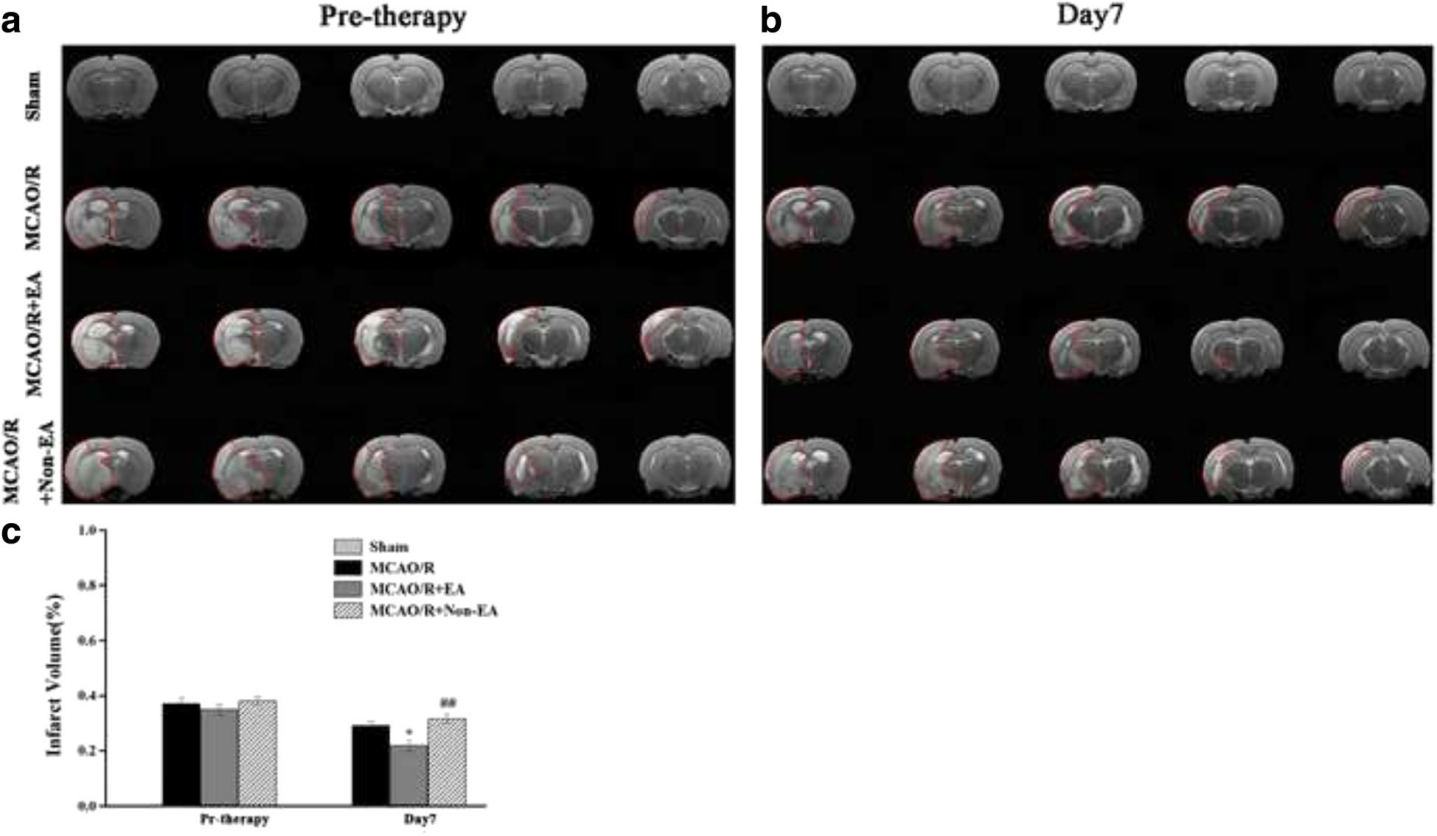
Effect of EA on cerebral infarct volume assessed by T2-Weighted MRI in Rats. **a**, **b** T2WI images of five brain slices of different levels (L10–L14 slices) from five different rats in each group before treatment and at Day 7 after EA. The ischemic lesion corresponds to the hyperintense region on each image. **c** The high intensity volume of T2WI calculated over all slices (infarct volume/volume of whole brains). Data are shown as mean ± S.E.M from 12 individual rats in each group. *P < 0.05, the MCAO/R + EA group versus the MCAO/R group; ^##^ *P* < 0.01, the MCAO/R + EA group versus the MCAO/R+ Non-EA group

To determine whether EA treatment could influence the development of ischemic infarct, T2WI was acquired before treatment and at Day 7 after EA. The results showed that in the Sham group, T2WI images were both intact hemispheres (Fig. 3a and b), and the high signal intensity volume of T2WI alterations in the ipsilateral hemisphere for damaged brains (representative 5 slices from the central infarct area) in the MCAO/R group, the MCAO/R + EA group and the MCAO/R+ Non-EA group before treatment and at Day 7 after EA (Fig. 3a and b). Quantitative analysis showed that the high signal intensity volumes on T2-Weighted MRI, which were corresponded to the infarct area, were decreased in the MCAO/R + EA group as compared with the MCAO/R group (*P* = 0.016, Fig. 3c) and the MCAO/R+ Non-EA group at Day 7 after EA treatment (*P* = 0.001, Fig. 3c). Meanwhile, the infarct lesion generally reduced on images acquired after treatment, as compared with that of before treatment (Fig. 3c). These results suggest that EA treatment can reduce the infarct volumes in the MCAO/R rats.

### EA treatment decreased cerebral astroglial and microglial/macrophage hyperplasia in peri-infarct area

**Fig. 4:**
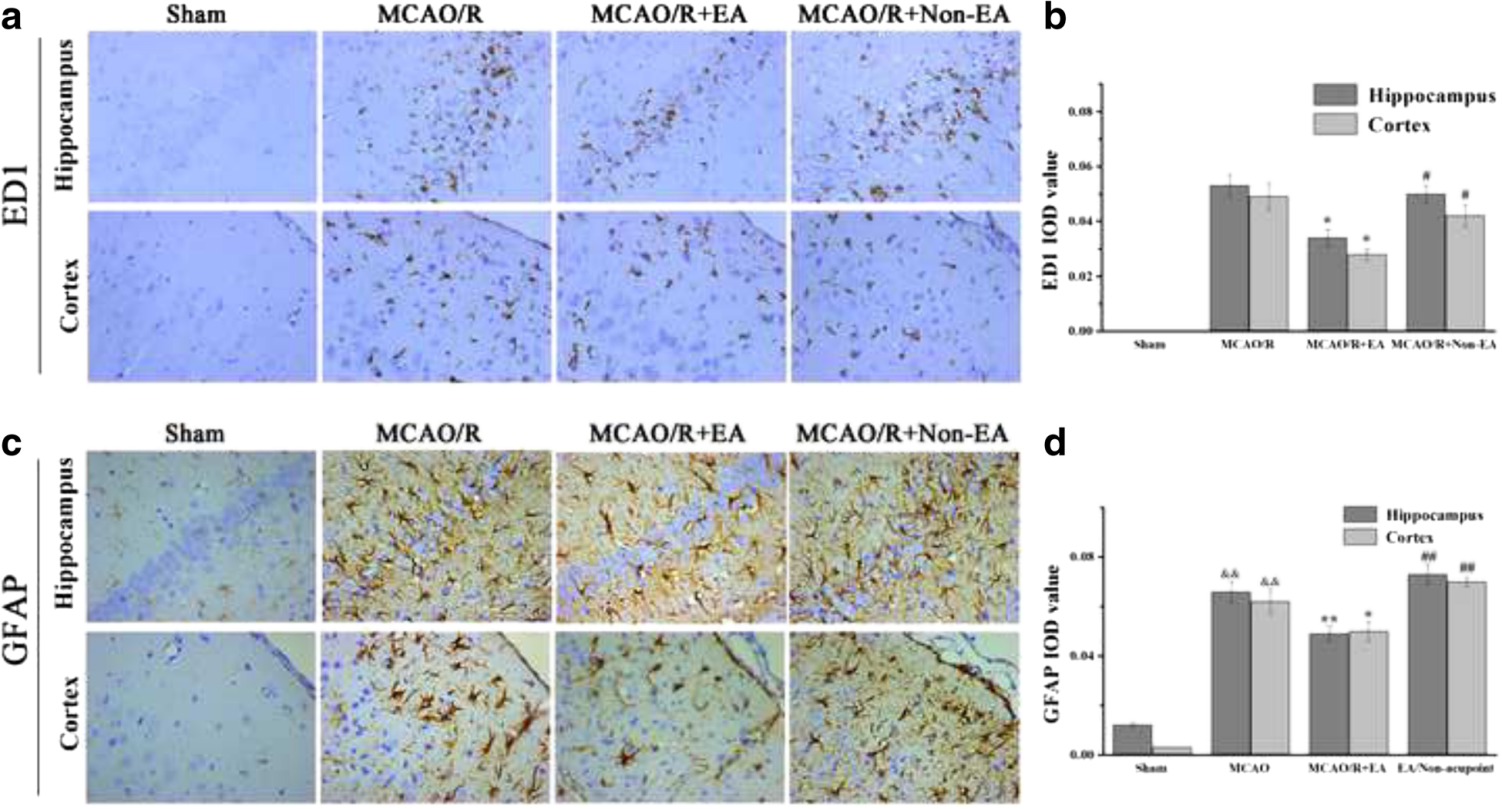
Effects of EA on astroglial and microglial/macrophage hyperplasia after MCAO/R injury. **a**, **c** Immunohistochemistry of ED1-positive and GFAP-positive cells in peri-infract hippocampal CA1 and sensorimotor cortex of each groups at Day 7 after EA treatment (brown cells, Scale bar = 20 μm). **b**, **d** Quantification of microglial/macrophage and astroglial positive cells showed lower density in the MCAO/R + EA group compared to the MCAO/R group and the MCAO/R+ Non-EA group. ED1-positive and GFAP-positive IOD in regions (0.3 × 0.2 mm^2^) were counted. Data are shown as mean ± S.E.M from 6 individual rats in each group. ^&&^ *P* < 0.01, the MCAO/R group versus the Sham group; **P < 0.01, *P < 0.05, the MCAO/R + EA group versus the MCAO/R group; ^##^ *P* < 0.01, ^#^ *P* < 0.05, the MCAO/R + EA group versus the MCAO/R+ Non-EA group

To investigate the influence of EA treatment on reactive glial cells, the ED1 protein, as a marker for activated microglia/macrophage, and the GFAP protein, as a marker for activated astrocytes, were assessed in sections of rat peri-infarct cerebral tissue. It remarkably increased in the peri-infarct hippocampal CA1 and sensorimotor cortex at Day 7 after EA (*P* < 0.01, Fig. 4a-d). However, ED1-positive cells of the MCAO/R + EA group were lower than the MCAO/R group (CA1: *P* = 0.031, cortex: *P* = 0.043, Fig. 4b) and the MCAO/R+ Non-EA group (CA1: *P* = 0.040, cortex: *P* = 0.028, Fig. 4b) at Day 7 after EA. GFAP-positive cells of the MCAO/R + EA group were lower than the MCAO/R group (CA1: *P* = 0.001, cortex: *P* = 0.023, Fig. 4d) and the MCAO/R+ Non-EA group (CA1: *P* < 0.001, cortex: *P* = 0.001, Fig. 4c) at Day 7 after EA. These results suggested that EA treatment could inhibit astroglial and microglial/macrophage hyperplasia in peri-infarct hippocampal CA1 and sensorimotor of MCAO/R rats.

### EA treatment attenuated inflammatory cytokine in peri-infarct hippocampal CA1 and sensorimotor cortex

**Fig. 5:**
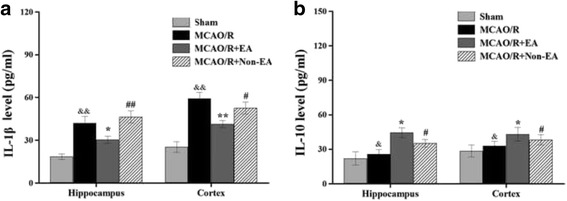
Effects of EA on inflammatory cytokines levels in the peri-infarct hippocampal CA1and sensorimotor cortex of MCAO/R rats. **a **Representative and quantitative analysis of ELISA showed that EA treatment for 7 days decreased the levels of IL-1β and **b** increased the levels of IL-10 in MCAO/R injured rats. Data are presented as mean ± S.E.M. from 6 individual rats in each group. ^&&^ *P* < 0.01, ^&^ *P* < 0.05, the MCAO/R group versus the Sham group; **P < 0.01 *P < 0.05, the MCAO/R + EA group versus the MCAO/R group; ^##^ *P* < 0.01, ^#^ *P* < 0.05, the MCAO/R + EA group versus the MCAO/R+ Non-EA group

Pro-inflammatory cytokines form gliosis such as IL-1β contribute to the detrimental post ischemic injury, by contrast, IL-10 as an anti-inflammatory cytokine is secreted by activated immune cells. The results revealed that the MCAO/R group rats showed elevated level of IL-1β (hippocampal: *P* < 0.001, cortex: *P* < 0.001, Fig. 5a) and IL-10 (hippocampal: *P* = 0.004, cortex: *P* = 0.010, Fig. 5b) in the peri-infarct hippocampal and sensorimotor cortex as compared with the Sham group (*P* ***<*** 0.05, Fig. 5a and b), and the MCAO/R + EA group rats showed a significantly lower level of IL-1β in comparison to the MCAO/R (hippocampal: *P* = 0.031, cortex: *P* = 0.003, Fig. 5a) and the MCAO/R+ Non-EA group (hippocampal: *P* = 0.004, cortex: *P* = 0.046, Fig. 5a) at Day 7 after EA (*P* ***<*** 0.05, Fig. 5a and b). However, EA increased the IL-10 level as compared with the MCAO/R group (hippocampal: *P* = 0.014, cortex: *P* = 0.044, Fig. 5b) and the Non-EA group (hippocampal: *P* = 0.018, cortex: *P* = 0.045, Fig. 5b), suggesting that EA treatment could attenuate the secretion of IL-1β, as well as promote the release of IL-10 to exert its anti-inflammatory effect.

### EA treatment decreased the astroglial and microglial/ macrophage P2X7R and P2Y1R in peri-infarct hippocampal CA1 and sensorimotor cortex

**Fig. 6:**
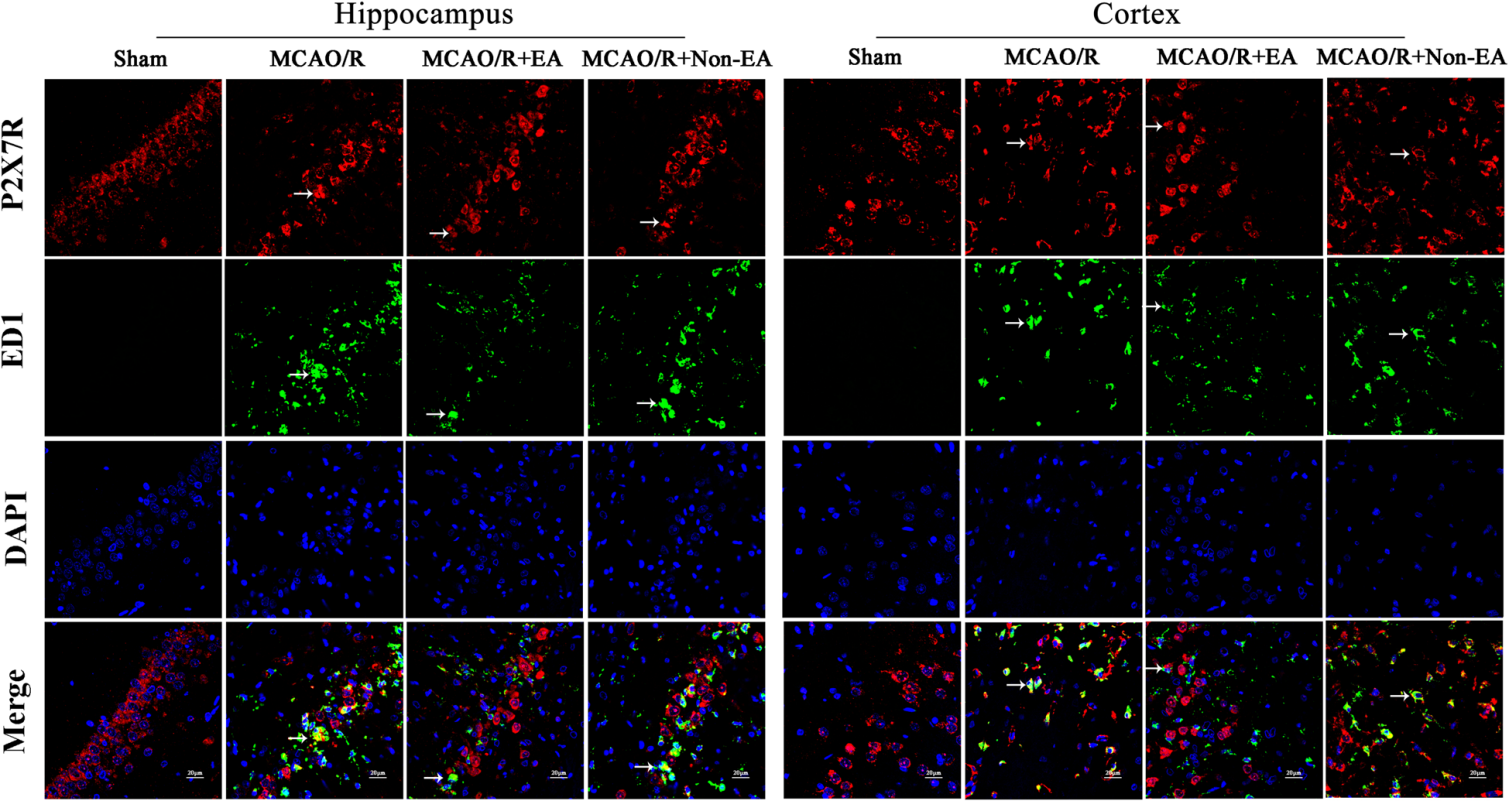
Effects of EA on P2X7R and ED1 co-expression after MCAO/R injury. The co-expression of P2X7R and ED1 was examined in the hippocampal CA1 and sensorimotor cortex of each groups at Day 7 after EA. P2X7R-positive cells are green. ED1-positive cells are red. P2X7R and ED1 double-positive cells are yellow. The arrows indicate the co-expression of P2X7R and ED1. Nuclei counterstained with DAPI (blue). Scale bar 20 μm

**Fig. 7:**
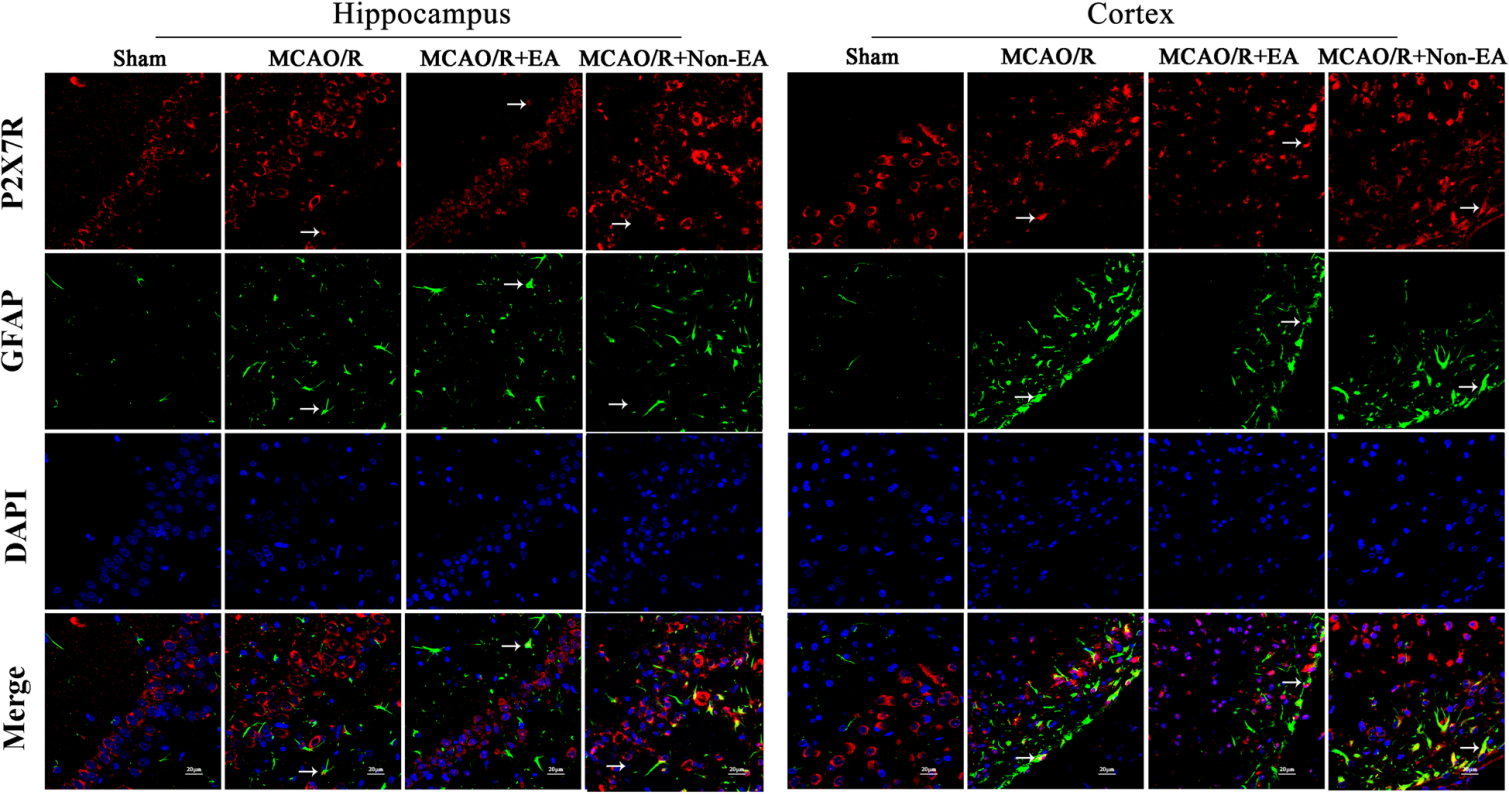
Effects of EA on P2X7R and GFAP co-expression after MCAO/R injury. The co-expression of P2X7R and GFAP was examined in the hippocampal CA1 and sensorimotor cortex at Day 7 after EA. P2X7R-positive cells are green. GFAP-positive cells are red. P2X7R and GFAP double-positive cells are yellow. The arrows indicate the co-expression of P2X7R and GFAP. Nuclei counterstained with DAPI (blue). Scale bar 20 μm

**Fig. 8:**
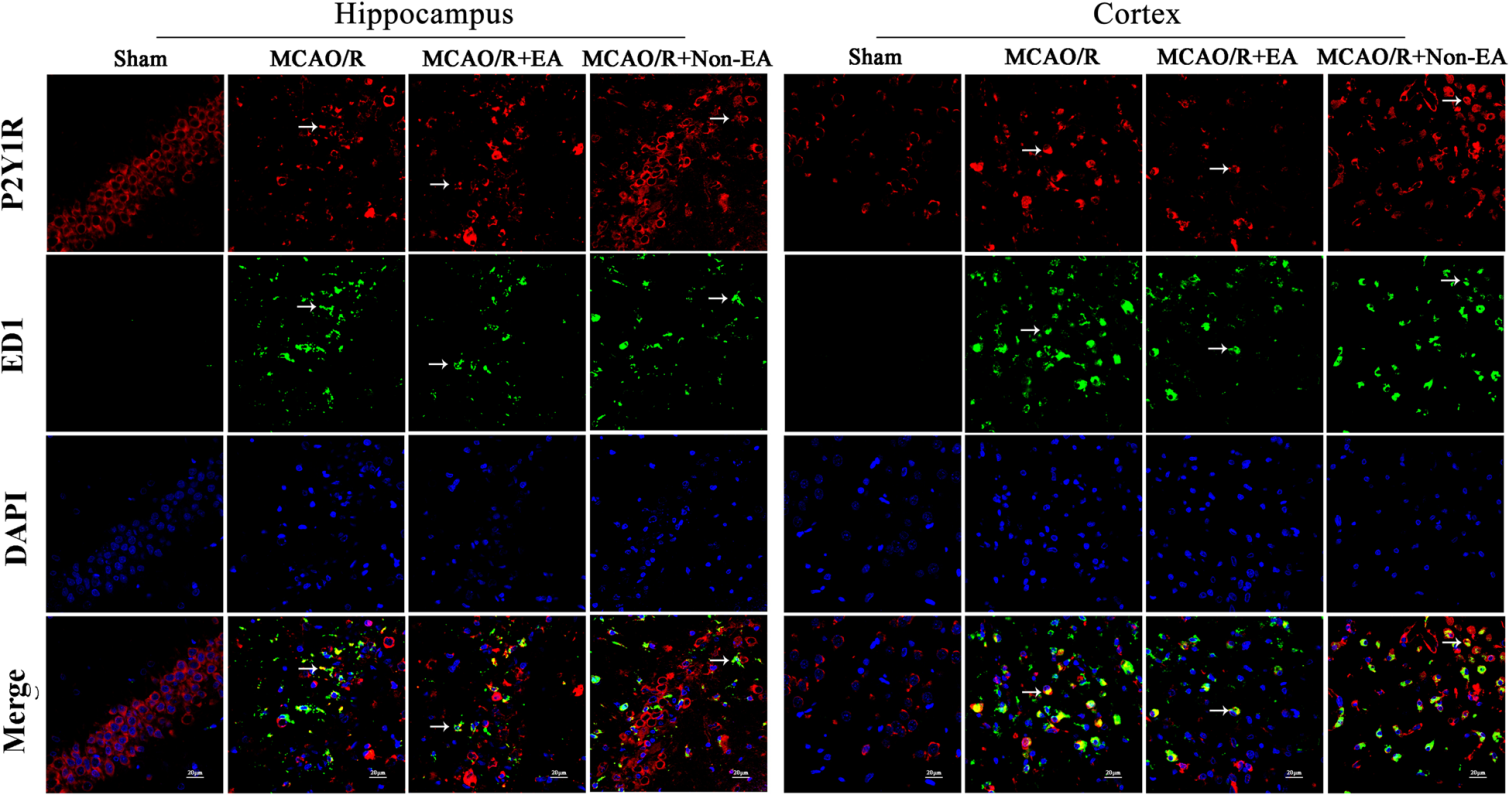
Effects of EA on P2Y1R and GFAP co-expression after MCAO/R injury. The co-expression of P2Y1R and GFAP was examined in the hippocampal CA1 and sensorimotor cortex at Day 7 after EA. P2Y1R-positive cells are green. GFAP-positive cells are red. P2Y1R and GFAP double-positive cells are yellow. The arrows indicate the co-expression of P2Y1R and ED1. Nuclei counterstained with DAPI (blue). Scale bar 20 μm

**Fig. 9:**
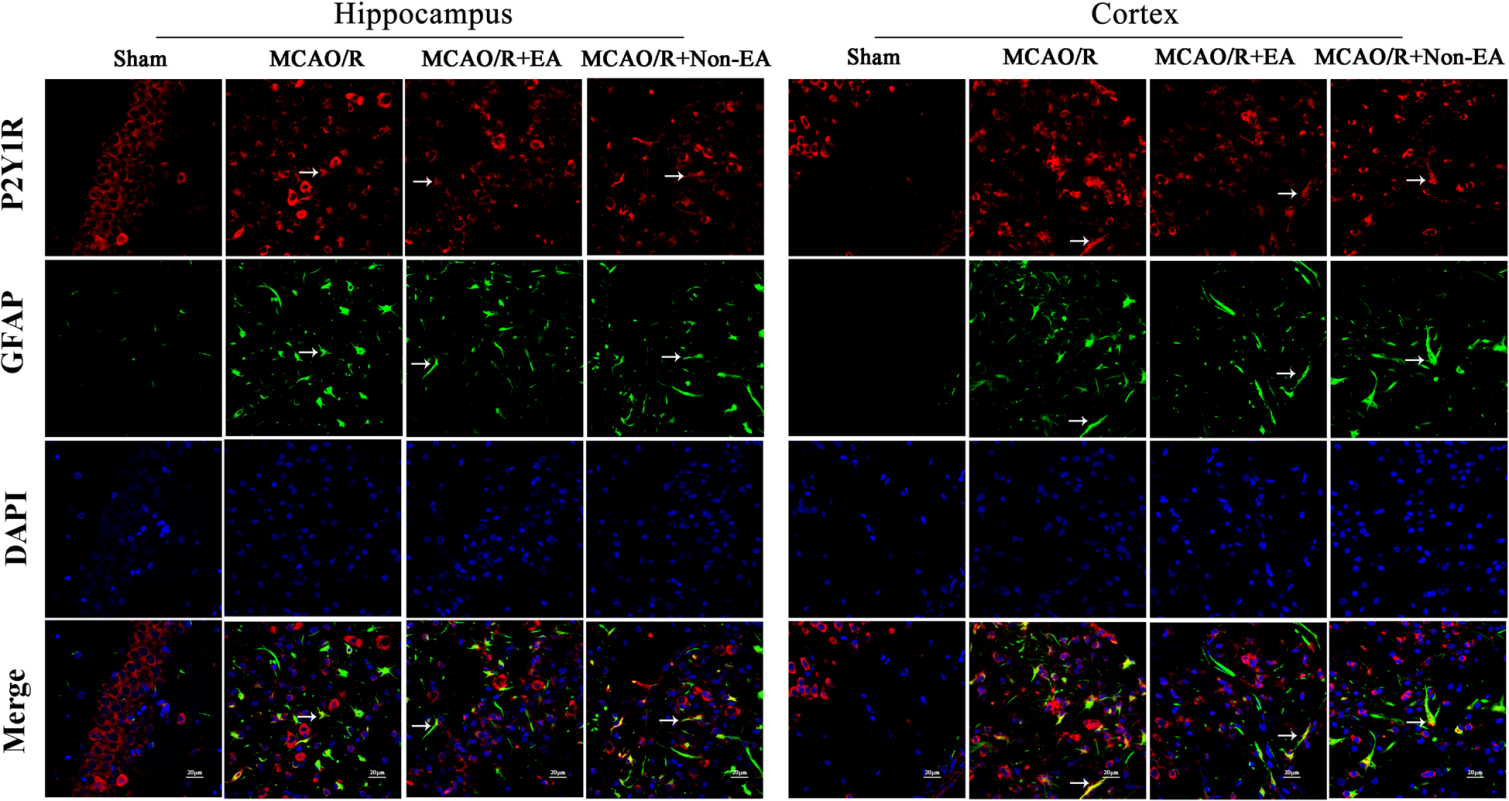
Effects of EA on P2Y1R and GFAP co-expression after MCAO/R injury. The co-expression of P2Y1R and GFAP was examined in the hippocampal CA1 and sensorimotor cortex at Day 7 after EA. P2Y1R-positive cells are green. GFAP-positive cells are red. P2Y1R and GFAP double-positive cells are yellow. The arrows indicate the co-expression of P2Y1R and GFAP. Nuclei counterstained with DAPI (blue). Scale bar 20 μm

**Fig. 10:**
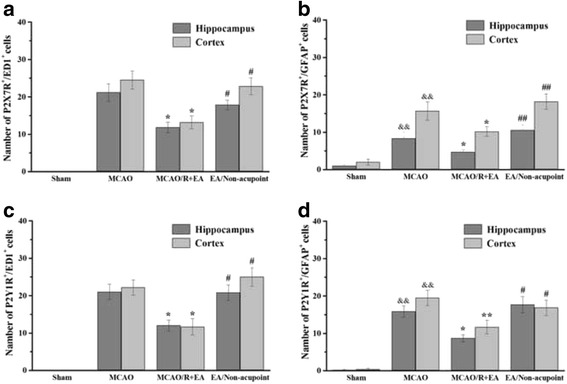
Quantification of co-expression in peri-infarct hippocampal CA1and sensorimotor cortex. Quantification of P2X7R and ED1(**a**), P2X7R and GFAP(**b**), P2Y1R and ED1 (**c**) and P2Y1R and GFAP (**d**) co-expression showed lower density in the MCAO/R + EA group compared to the MCAO/R group and the MCAO/R+ Non-EA group in peri-infarct sensorimotor cortex. Quantification of P2X7R and ED, P2Y1R and ED1 and P2Y1R and GFAP co-expression showed lower density in the MCAO/R + EA group compared to the MCAO/R group and the MCAO/R+ Non-EA group in peri-infarct hippocampal CA1. Data are shown as mean ± S.E.M from 6 individual rats in each group. ^&&^ *P* < 0.01, the MCAO/R group versus the Sham group; **P < 0.01, *P < 0.05, the MCAO/R + EA group versus the MCAO/R group; ^##^ *P* < 0.01, ^#^ *P* < 0.05, the MCAO/R + EA group versus the MCAO/R+ Non-EA group

Furthermore, to investigate the mechanism of EA-inhibited astroglial and microglial/macrophage hyperplasia and inflammation, the location expression of P2X7R and P2Y1R were analyzed. The results showed that the co-expression of P2X7R and ED1, P2X7 and GFAP, P2Y1R and ED1, P2Y1R and GFAP occurred in peri-infarct hippocampal CA1 and sensorimotor cortex in the MCAO/R group, the MCAO/R + EA group and the MCAO+ Non-EA group compared to the Sham group (Figs. 6, 7, 8 and 9). The co-expression of P2X7R^+^/ED1^+^ was significantly decreased in the MCAO/R + EA group compared with the MCAO/R group (CA1: *P* = 0.036, cortex: *P* = 0.017, Fig. 10a) and the MCAO/R+ Non-EA group (CA1: *P* = 0.044, cortex: *P* = 0.030, Fig. 10a). The co-expression of P2X7R ^+^/GFAP^+^ was significantly decreased in the MCAO/R + EA group compared with the MCAO/R group (CA1: *P* = 0.040, cortex: *P* = 0.039, Fig. 10b) and the MCAO/R+ Non-EA group (CA1: *P* = 0.002, cortex: *P* = 0.004, Fig. 10b). The co-expression of P2Y1R^+^/ED1^+^ was significantly decreased in the MCAO/R + EA group compared with the MCAO/R group (CA1: *P* = 0.027, cortex: *P* = 0.023, Fig. 10c) and the MCAO/R+ Non-EA group (CA1: *P* = 0.030, cortex: *P* = 0.011, Fig. 10c). The co-expression of P2Y1R^+^/GFAP^+^ was significantly decreased in the MCAO/R + EA group compared with the MCAO/R group (CA1: *P* = 0.013, cortex: *P* = 0.004, Fig. 10d) and the MCAO/R+ Non-EA group (CA1: *P* = 0.028, cortex: *P* = 0.046, Fig. 10d). These results suggested that EA treatment could markedly alleviate astroglial and microglial/ macrophage P2X7R and P2Y1R in peri-infarct hippocampal CA1 and sensorimotor cortex in rats that underwent MCAO/R brain injury.

## Discussion

Ischemic stroke is one of the most common causes of mortality and complex disability in adults worldwide. Approximately 70% of stroke survivors suffer from motor impairment, 25% of patients present with cognitive impairment 3 months after a stroke [33, 34]. Up to 65% of people with stroke may be considered to have cognitive impairment when selective types of cognitive impairment, commonly involving memory, orientation, language and attention [35]. Cognitive impairment is a critical mental deficit that severely affects the quality of life among people with stroke. Neuro-inflammation is a crucial element in ischemic cascade that result in key brain areas nerve injury or death contributing the following motor and cognitive impairments [36, 37]. After ischemic stroke onset, gliosis, microglia, the resident immune cells of CNS, are activated within minutes, reaching a peak at 6–12 h, decreasing at several days after initial injury [38]. In addition, under ischemic stroke, ATP is released from damaged neurons or glia cells of infarct core region to extracellular space invoking peri-infarct astroglial and microglial/macrophage membrane P2 purinoceptors signaling, promoting the nucleus translocation of NF-κB-p65 to induce pro-inflammatory cytokine transcription and secretion, which is likely to initiate or aggravate motor and cognitive impairment [8, 13, 14].

Acupuncture has been used in the treatment of stroke for thousands of years in China. And EA, engrafted electric stimulation to the acupoints through acupuncture needles, has been widely used in clinical practice for the treatment of motor, cognition, sensation and other neurological dysfunctions in people with stroke [39]. Simultaneously, accumulating evidences have demonstrated that EA can alleviate the inflammatory responses in cerebral ischemic stroke [40]. The current study demonstrated that EA at the DU 20 and DU 24 acupoints treatment from the 12 h after MCAO/R injury for consecutive 7 days effectively reduced cerebral infarct and pro-inflammatory cytokine release accompanied by the improved neurological deficit as well as the motor and memory impairment outcomes.

To elucidate the underlying anti-neruoinflammation mechanisms of EA-treated MCAO/R injury, the P2 purinoceptors alterations in hippocampal CA1 and sensorimotor cortex were identified. P2 purinoceptors are sporadically scattered in almost all areas of the brain including cerebral cortex, hippocampus. It has also been identified on microglia, astrocytes, oligodendrocytes and neurons [41, 42]. Increasing evidences have shown that the P2 purinoceptors is playing an important role in neuroinflammation. After an ischemic insult, microglia is rapidly activated via stimulation of P2X7R and P2Y1R by high concentrations of ATP released from damaged neurons and other glial cells, which promotes production and secretion of inflammatory mediators [43, 44]. In addition, P2X7R plays a key role in IL-1β secretion from reactive microglial cells [45]. The LPS-induced inflammatory injury models were recently proposed to explain the secretion of mature IL-1β following P2X7R activation [46]. Studies have suggested that activated astrocytes increased the expression of P2Y1R to promote pro-inflammatory cytokines release in vivo or in vitro [47]. There is increasing evidence that P2Y1R plays a role in astrocytes/macrophages–mediated neuroinflammation, which could lead to positively regulate transcription of inflammatory genes, such as TNF-α, IL-1β following post-stroke cognitive impairment [48]. Conversely, inhibiting of P2X7R obviously reduced ischemia and reperfusion-induced neuronal death by decreasing the expression levels of IL-1ß, TNF-α and IL-6 in the hippocampus, and increasing survival rates [49]. BBG, a P2X7R antagonist, can reduce MCAO/R injury-induced microglial microvesicle-like components, IL-1β expression, glial activation and delayed neuronal death in hippocampal CA1 region after ischemic stroke [50]. 2-MeSADP, a P2Y1R inhibitor, could protect LPS-induced TNF-α release in cortical astrocyte cells [51]. MRS2179, a P2Y1R antagonist, by using lateral cerebral ventricle injection, could reduce the MCAO/R injury-induced infract volume and decrease the mRNA expression of IL-6, TNF-α, MCP-1 and increase IP-10 level in rats, as wells as block down astroglial cellular activation in vitro. Therefore, it would be noteworthy that inhibitions of astroglial and microglial/macrophage-mediated signaling pathway could decrease the astrocytes-mediated neuroinflammation following cerebral ischemia.

As for how electroacupuncture attenuate ATP release in ischemic condition. ATP is an excitatory neurotransmitter in many synapses, acting as a co-transmitter in both the peripheral and CNS [16, 52]. It released from dying cells is likely to be a secondary mechanism to worsen the extent of the damage in cerebral ischemia [53]. Extracellular ATP can activate the family of ionotropic (P2XR) and metabotropic (P2YR) receptors to serve as neurotransmitter, neuromodulator, neuromodulator, astrocyte-to-neuron gliotransmitter and a controller of the chemotaxis [54, 55]. Thus, brain energy deprivation causes anoxic depolarization which, if prolonged, leads to irreversible neuronal and glial death [56]. In peripheral nerves, purinergic receptor serve as a message transmit passes EA stimulus signals from peripheral sensory nerves to cross talked cerebral center. Thus, EA induces the release of extracellular ATP in turn activates purinergic receptors on the sensory nerves of the skin, then transmits those messages to the spinal cord nerve root and higher brain centers that control autonomic functions and modulate neural activities [4–7]. In our study, EA treatment at the DU20 and DU24 acupoints obviously inhibited astroglial and microglial/macrophage hyperplasia and the levels of P2X7R and ED1, P2X7 and GFAP, P2Y1R and ED1, P2Y1R and GFAP co-expression in peri-infarct hippocampal CA1 and sensorimotor cortex compared with that of MCAO/R model and Non-EA treatment, which demonstrated that astroglial and microglial/macrophage P2 purinoceptors-mediated neuroinflammation and hyperplasia in peri-infarct hippocampal CA1 and sensorimotor cortex were attenuated by EA treatment after ischemic stroke accompanied by the improved motor and memory behavior performance. According to the results, EA may suppress extracellular ATP invoked purinergic receptors signaling to inhibit pro-inflammatory cytokines release following cerebral ischemia. Similarly, published data showed that EA treatment relieved pain hypersensitivity via the down-regulation of spinal microglial P2X7R-mediated overexpression of IL-1β.

## Conclusion

In conclusion, our data demonstrated the hypothesis that EA could exert its anti-inflammatory effect via inhibiting the astroglial and microglial/macrophage P2 purinoceptors-mediated neuroinflammation after MCAO/R injury. In addition, the study would aim to elucidate the precise mechanisms of EA on astroglial and microglial P2X7R using P2X7R knock-out and knock-in mice without or with ischemic stroke.

## Additional file

Additional file 1: A time-line diagram was attached in the supplementary file which demonstrated the time points of each experimental step, including: time for modeling, neural assessments, treatment period, water maze test and lab indexes testing. (PDF 10 kb)

## Abbreviations

ATP: Adenosine 5′-triphosphate
CNS: Central nervous system
EA: Electroacupuncture
ELISA: Enzyme-linked immunosorbent assay
IL-1β: Interleukin-1β
IL-6: Interleukin-6
IS: Ischemic stroke
MCAO/R: Middle cerebral artery occlusion and reperfusion
mNSS: modified neurological severity scores
NF-κB: Nuclear factor kappa b
P2X7R: P2X purinoceptor 7
P2Y1R: P2Y purinoceptor 1
T2WI: T2-weighted images
