# Supplementary figures and images for: Electroacupuncture ameliorating post-stroke cognitive impairments via inhibition of peri-infarct astroglial and microglial/macrophage P2 purinoceptors-mediated neuroinflammation and hyperplasia
Source: BMC Complement Altern Med. 2017 Oct 10;17:480. doi: 10.1186/s12906-017-1974-y (PMC5635586; doi:10.1186/s12906-017-1974-y)

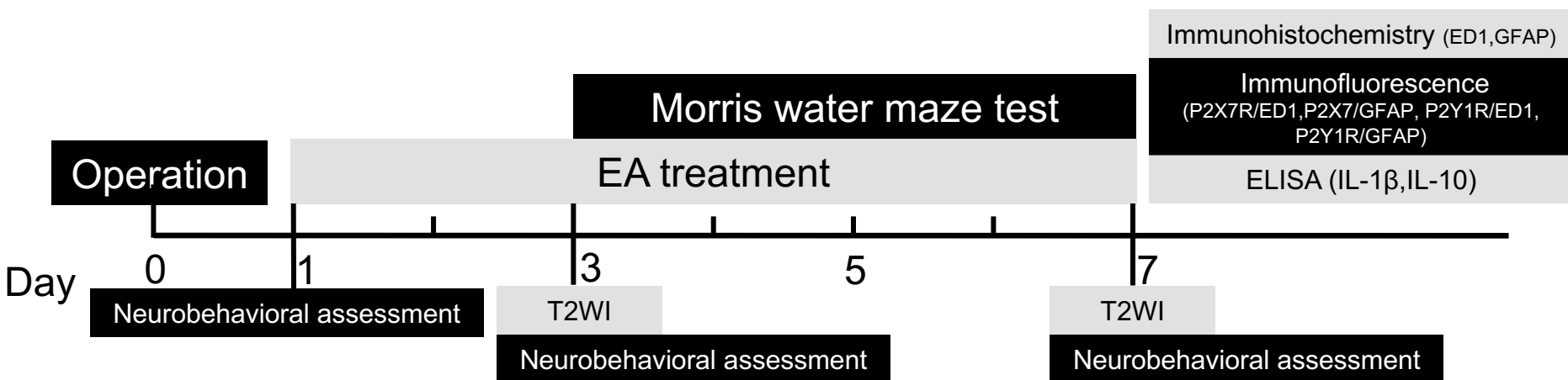

Supplement: Additional file 1: — A time-line diagram was attached in the supplementary file which demonstrated the time points of each experimental step, including: time for modeling, neural assessments, treatment period, water maze test and lab indexes testing. (PDF 10 kb) [file 12906_2017_1974_MOESM1_ESM.pdf]
